# Spinal Infection Due to Enterococcus faecalis as the First Manifestation of Colorectal Cancer

**DOI:** 10.7759/cureus.39815

**Published:** 2023-06-01

**Authors:** Eni Manoku, Guilherme S Piedade, Andreas L Gelhardt, Joacir G Cordeiro, Jorge A Terzis

**Affiliations:** 1 Department of Neurosurgery, Helios Universitätsklinikum Wuppertal, Wuppertal, DEU; 2 Department of Neurosurgery, Medical School, Federal University of Paraná, Curitiba, BRA; 3 Department of Neurological Surgery, University of Miami, Coral Gables, USA

**Keywords:** colorectal cancer, hematogenous dissemination, enterococcus faecalis, spondylitis, spinal epidural abscess

## Abstract

Spinal epidural abscess is a relatively infrequent surgical indication, but it may be neurologically compromising. The most frequent pathogen is *Staphylococcus aureus*, present in two-thirds of the cases. *Enterococcus faecalis* is part of the intestinal flora and is uncommon in this condition. Colorectal cancer is reported to be a cause of hematogenic translocation and distant infection. We present a case of an 82-year-old patient admitted for acute low back pain with increased inflammatory markers and negative blood cultures. An MRI revealed an epidural lumbar abscess with adjacent spondylitis. After surgical treatment, *E. faecalis* was identified, and antibiotics were adjusted accordingly. A colonoscopy revealed colon cancer. This is the first case in the literature of a spinal epidural abscess by *E. faecalis* as the first manifestation of a newly diagnosed colorectal cancer. When facing a spinal infection caused by atypical intestinal bacteria and no other clear sources, a colonoscopy should be considered.

## Introduction

Spinal epidural abscess is a severe but relatively infrequent condition in European neurosurgical practice [1]. It affects patients from every age group but is more often seen in adults in their fifth and sixth decades of life and in younger subjects with intravenous drug abuse [2]. The most common presenting symptom is spontaneous back pain, possibly accompanied by fever and neurological deficits. Those deficits are usually rapidly progressive due to the spinal cord or cauda equina compression. Known risk factors are intravenous drug abuse, HIV infection, immunosuppression, previous spinal surgery, trauma, diabetes mellitus, and concomitant infection [3].

The most frequent pathogen is *Staphylococcus aureus*, present in two-thirds of the cases [1]. It is followed by gram-negative bacilli and *Streptococcus* species [4]. We present a case of lumbar epidural abscess caused by *Enterococcus faecalis*, a member of the intestinal flora that is much less common [5-9]. The association of some hematogenic infections with colon cancer is well described in the literature. For instance, endocarditis secondary to *Streptococcus bovis* classically requires a colonoscopy due to this notable association [10]. In this report, we describe an epidural abscess due to *E. faecalis* as the very first manifestation of colorectal cancer, and, to the best of our knowledge, it is the first case in the literature.

## Case presentation

**Figure 1:**
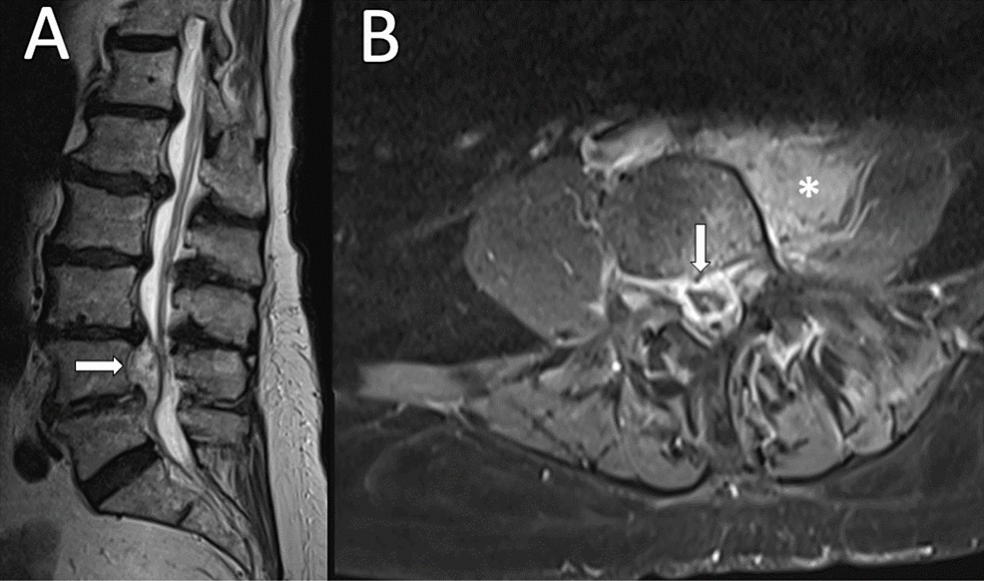
MRI revealed an epidural abscess A: T2 imaging displaying the epidural abscess. B: Axial T1 with contrast evidencing the epidural component (arrow) and the psoas abscess (*).

**Figure 2:**
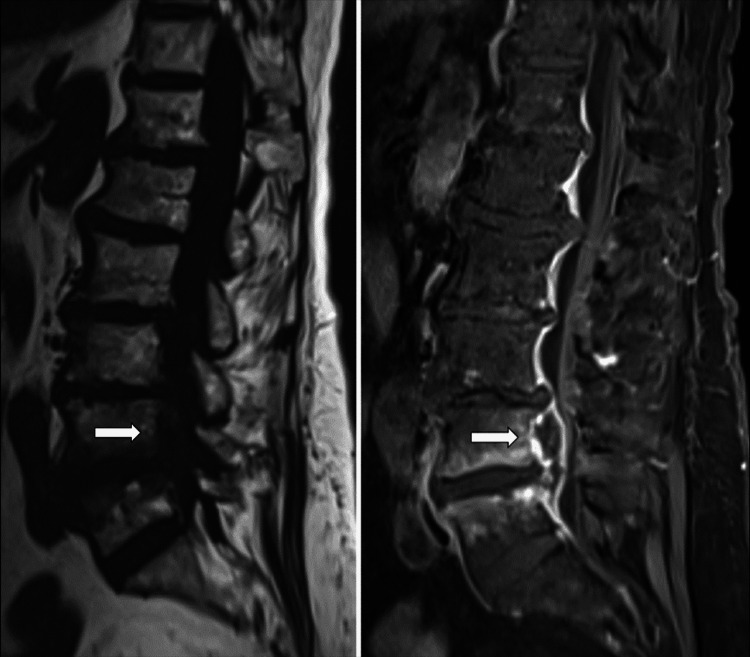
MRI with and without contrast evidenced the epidural abscess and spondylitis

We report the case of an 82-year-old female who presented to the ER of an outside hospital complaining of severe low back pain for one week. The physical exam was unremarkable, and the patient had a past medical history of atrial fibrillation and lumbar decompression for spinal stenosis at L3/4 done eight years prior. She had increased inflammatory markers (C-reactive protein (CRP) = 11 mg/dl; WBC = 12.900/µl). Blood cultures were negative. Routine infectious screening was negative and empirical piperacillin/tazobactam was started. MRI of the lumbar spine was performed three days later. It pointed to an epidural collection from L3 to S1, compatible with epidural abscess and spondylitis. Adjacent soft-tissue edema and extensive contrast enhancement in the intervertebral compartment, paravertebral soft tissues, and psoas muscle were observed (Figures 1, 2).

Empiric antibiotic therapy was adjusted to flucloxacillin given the infection location. The back pain worsened, and she presented with melena. Complete blood count pointed to normocytic anemia (hemoglobin = 9 g/dL, mean corpuscular volume = 80 fL, mean corpuscular hemoglobin = 27 pg, RBC = 3.43 x 106/µl). The digital rectal exam and fecal occult blood test were positive. The colonoscopy showed a mass in the left colon flexure over a length of 5 cm with resulting stenosis and the biopsy confirmed colorectal cancer. The contrasted CT of the abdomen ruled out a possible fistula. The patient was referred to our department of neurosurgery with increasing pain four days after the spinal MRI. A lumbar hemilaminectomy 4/5 with the evacuation of the epidural empyema was performed.

Afterward, the back pain markedly improved. The patient was neurologically intact throughout the admission. The intraoperative culture swabs revealed *E. faecalis*. Piperacillin/tazobactam was resumed and confirmed to be appropriate by the sensitivity profile.

On the second postoperative day, the pain subsided completely. The patient was transferred back for further conservative management and oncological workup with the recommendation of a short-term lumbar spine MRI follow-up.

## Discussion

*E. faecalis* is rarely a cause of spinal epidural abscess. This gram-positive bacterium inhabits the gastrointestinal tract of healthy individuals and can reach the spinal canal via a hematogenous route, direct extension, or direct inoculation. Cone et al. described cases of spinal epidural abscess with *E. faecalis* following subacute endocarditis [9]. Alpantaki et al. reported an epidural abscess associated with an epidural catheter used for anesthesia [8]. Lee et al. published the case of an epidural abscess with *E. faecalis* caused by an epidural steroid injection [6]. Most cases will remain, however, without an identifiable source.

There is a classic association between colorectal cancer and endocarditis by *Streptococcus bovis* [10]. Reports support the association between this malignancy and the translocation of *E. faecalis* [11,12]. Pericàs et al. reported an interesting cohort of 154 patients with endocarditis by *E. faecalis*, 109 of them without an identifiable source. Half of this subgroup underwent colonoscopy and 50.8% had some form of colorectal neoplasm [13]. For this reason, the authors advocate for colonoscopy in cases of infection by *E. faecalis* without a clear source. It is hypothesized that this pathogen is linked to mutagenesis in colonic epithelial cells, causing lesions that lead to its translocation to the bloodstream [14].

In the presented case, direct spreading by adjacent structures was ruled out in the CT of the abdomen. A plausible pathophysiological mechanism for our patient remains the hematogenic dissemination, despite negative blood cultures. *E. faecalis* is part of the normal intestinal flora and can translocate through the intestine, especially when the layering is disrupted by neoplasm or other injuries. For that reason, we agree with Pericàs et al. and find reasonable the indication for colonoscopy for infections with *E. faecalis* in the absence of an identifiable source.

*S. aureus* is the most common culprit for epidural abscesses. Flucloxacillin has in vitro activity against gram-positive and gram-negative aerobic and anaerobic bacteria; however, using it as monotherapy did not result in source control. Retrospectively, we consider it would have been more adequate to continue empiric broad spectrum coverage until speciation was achieved. The presence of an epidural abscess may represent an indication for surgery. Conservative management may be indicated in small abscesses, absence of sepsis, neurologically intact and oligosymptomatic patients, high surgical risk, and/or holospinal epidural collections. In case of negative blood cultures, CT-guided sampling could be a viable option.

In the reported case, the patient was taken to the OR given symptomatic worsening despite IV antibiotics. The evacuation was readily effective for pain, source control, and agent identification. Some could argue that the presence of *E. faecalis* and colon cancer were coincidental. Nevertheless, based on other reported associations of cancer and this infection and given it is part of routine preventive care, we recommend that colonoscopy should be considered in case of spinal infections caused by atypical intestinal bacteria with no other clear sources.

## Conclusions

This is the first case in the literature of a spinal epidural abscess by *E. faecalis* as the first manifestation of a newly diagnosed colorectal cancer. Hematogenous dissemination is the accepted pathway. When facing a spinal infection caused by atypical intestinal bacteria and no other clear sources, a colonoscopy should be considered.
